# Adult-type granulosa cell tumor associated with elevated luteinizing hormone: Two rare case reports

**DOI:** 10.1097/MD.0000000000037069

**Published:** 2024-02-16

**Authors:** Yujing Wang, Na Wang, Xuejiao Zhang, Zijie Fu, Chao Pang, Yuan Zhang, Xiaodong Li

**Affiliations:** a Department of Gynecology, The First Hospital of Hebei Medical University, 050030, Shijiazhuang, China; b Department of Pathology, The First Hospital of Hebei Medical University, 050030, Shijiazhuang, China; c Department of Gynecology, The Second Hospital of Hebei Medical University, 050004, Shijiazhuang, China

**Keywords:** Adult-type granulosa cell tumor of the ovary, case report, inhibin B, luteinizing hormone elevation, testosterone

## Abstract

**Introduction:**

Adult-type granulosa cell tumors (AGCTs), which account for 2% to 5% of all malignant ovarian tumors, are rare sex cord-stromal tumors that usually secrete excess estrogens, but they can also secrete androgens.

**Patient concerns:**

We report 2 patients of childbearing age with AGCT who presented with the complaint of abnormal menstruation and elevated luteinizing hormone (LH), and mildly elevated testosterone.

**Diagnosis:**

The ovarian tumors had hormonal activity.

**Interventions:**

The 2 patients underwent laparoscopic left adnexectomy. The second patient underwent 4 cycles of chemotherapy with paclitaxel and carboplatin as adjuvant treatments.

**Outcomes:**

Their postoperative pathology confirmed AGCTs. Also, their menstrual cycle returned to normal, with normal serum LH and testosterone levels. There was no sign of recurrence.

**Conclusion:**

The cases suggest that elevated serum LH levels may be a sign of unknown tumors in cases of oligomenorrhea or secondary amenorrhea. It is useful to evaluate the serum levels of inhibin B and anti-Müllerian hormone to improve the early recognition of ovarian granulosa cell tumors.

## 1. Introduction

Granulosa cell tumors (GCTs) of the ovary are sex cord-stromal tumors that originate from the granulosa cell layer of the ovarian follicles.^[1]^ GCTs are divided into adult- and juvenile-type tumors, based on clinical and pathological findings.^[2]^ Adult-type GCTs (AGCTs) constitute 95% of all ovarian GCTs and are the most common malignant sex cord-stromal tumors.^[2]^ AGCTs can occur in women of any age, but they are primarily diagnosed in perimenopausal and early postmenopausal women, with a peak at 50 to 54 years of age.^[3]^AGCTs are steroid-secreting tumors; most secrete estrogens, whereas <3% secrete androgens.^[4]^ To our knowledge, 5 reports have detailed elevated luteinizing hormone (LH) levels in adult-type ovarian GCTs (Table 2). Here, we report 2 patients with histologically confirmed AGCT who presented with abnormal menstruation and elevated LH, and mildly elevated testosterone (T), and with normal and normal follicle-stimulating hormone (FSH), and estradiol (E2) levels. We also present a review of relevant literature.

## 2. Case presentation

### 2.1. Case A

A 38-year-old married Chinese woman presented to our gynecology clinic with a complaint of secondary amenorrhea for >1 year. She had gained 10 kg during the previous 6 months, with a body mass index of 24.4 kg/m^2^. Hirsutism or virilization was observed. She was 13 years old at menarche and had experienced regular menstrual cycles of 28 days until her most recent pregnancy.

At 2.5 months after the last induced abortion, the patient’s menstrual cycle had not recovered; thus, the patient presented to a local hospital. She was prescribed oral progesterone capsules (200 mg/day for 10 days), but no withdrawal bleeding occurred. Withdrawal bleeding was subsequently induced by the sequential administration of estrogen and progestin.

Serum hormone assays revealed an elevated LH level (25.22 mIU/mL) but normal FSH, E2, progesterone, T, and prolactin levels (Table 1). Repeated serum hormone assays revealed ongoing and increasingly elevated LH levels (reaching 31.81 mIU/mL; Table 1).

**Table 1:** The patients’ serum sex hormone profiles before surgery.

	Case A				Case B
	March 23, 2021	May 12, 2021	August 30, 2021 (after taking oral contraceptives)	November 5, 2021	
LH (mIU/mL)	*25.22*	*31.81*	*4.24*	*37.44*	*19.08*
FSH (mIU/mL)	3.04	3.09	0.52	5.02	7.12
T (ng/mL)	0.39	0.20	0.13	*0.85*	*0.93*
E2 (pg/mL)	31.48	28.35	5	25.61	36.14
P (ng/mL)	1.88	1.45	0.58	2.61	0.69
PRL (ng/mL)	10.86	10.86	11.28	11.73	4.15

E2 = estradiol, FSH = follicle-stimulating hormone, LH = luteinizing hormone, P = progesterone, PRL = prolactin, T = testosterone

The patient was diagnosed with polycystic ovary syndrome (PCOS) and prescribed oral contraceptive tablets (spironolone ethinylestradiol) with monthly withdrawal bleeding for 3 months. Consequently, the patient’s LH level decreased to 4.247 mIU/mL (Table 1).

Transvaginal ultrasonography revealed a 2.9 × 1.9-cm cystic mass originating from the left ovary. The patient failed to maintain a normal menstrual cycle without medication. Repeated serum hormone assays revealed elevated LH (37.44 mIU/mL) and T (0.85 ng/mL) levels (Table 1). However, the patient’s tumor marker levels were within the normal limits.

**Figure 1.**
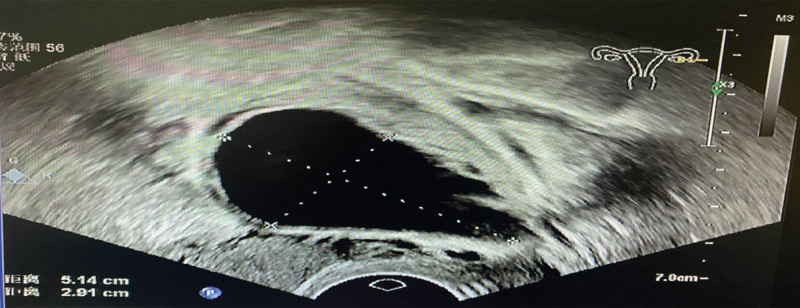
Transvaginal ultrasonography (case A) revealed a 5.1 × 2.9-cm cyst in the left ovary.

During treatment, the patient’s left ovarian cyst grew gradually, reaching a size of 5.1 × 2.9 cm on repeated ultrasonography within 3 months (Fig. 1). These findings suggested that the left ovarian cyst had hormonal activity. Therefore, surgery was recommended; however, the patient refused surgery.

Seven weeks later, repeated transvaginal ultrasonography revealed a cystic mass of 6.8 cm in diameter in the left ovary. The uterus was normal and the endometrial thickness was 0.48 cm. Blood examination revealed a markedly elevated serum anti-Müllerian hormone (AMH) level of 13.18 ng/mL. The patient and her family agreed to surgery.

Exploratory laparoscopy confirmed the presence of a left ovarian tumor with a well-defined capsule and no ascites. There was no evidence of extraovarian disease; thus, a left salpingo-oophorectomy was performed.

**Figure 2.**
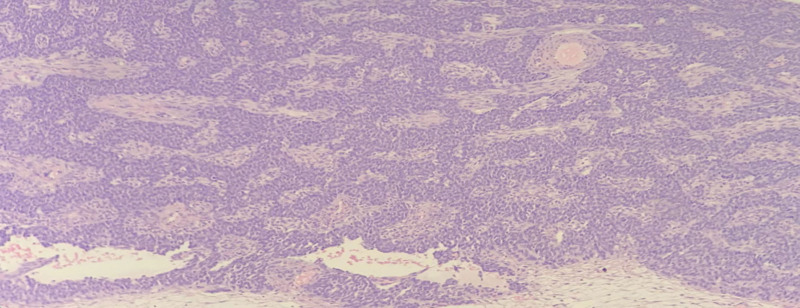
Pathological findings of the AGCT in case A (38-year-old woman, left ovary) (arrow: Call-Exner bodies). AGCT = adult-type granulosa cell tumor (100×).

Macroscopically, the resected ovarian tumor was cystic, yellow, and 7 cm in diameter. Histopathological testing revealed AGCT with cystic degeneration (Fig. 2). Immunohistochemical analysis showed that the tumor cells expressed FOXL2 and a genetic mutation, FOXL2 c.402C>G (p.C134W), was subsequently found.

An elevated serum inhibin B level of 187.64 pg/mL was detected 3 days after surgery. Based on the histopathological findings of the GCT, we suggested secondary laparoscopy with staging. The patient was unwilling to undergo a second surgical procedure. The patient’s postoperative course was uneventful.

Spontaneous menstruation occurred 3 weeks after surgery. The patient’s LH, T, inhibin B, and AMH levels returned to normal after surgery. Regular menstrual cycles were established, and there was no evidence of recurrence at 1-year follow-up.

### 2.2. Case B

A 25-year-old married Chinese woman complained of oligomenorrhea for >10 months and was referred to our clinic. She had experienced menarche at 12 years of age, and her menstrual cycle had become irregular (range: 1.5–2 months) 10 months earlier. No signs of virilization were apparent. The patient’s body mass index was 26.15 kg/m^2^.

**Figure 3.**
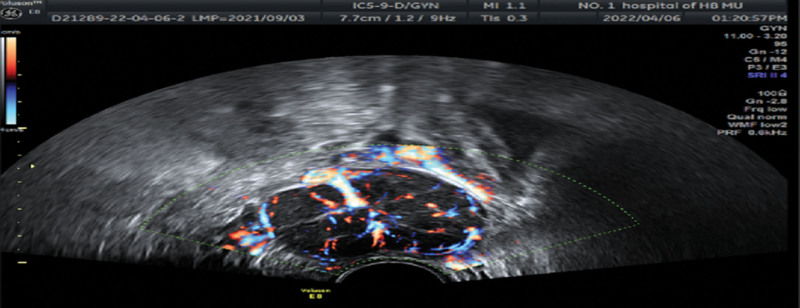
Transvaginal ultrasonography (case B) revealed a 4.6 × 3.5 × 3.3-cm solid mass with increased peripheral vascularity in the left ovary.

Pelvic examination revealed a solid mass in the left adnexal area, which was 5 cm in diameter, freely mobile, and non-tender. Transvaginal ultrasonography revealed a 4.6 × 3.5 × 3.3-cm solid mass with increased peripheral vascularity in the left ovary (Fig. 3); the uterus was normal in size with an endometrial thickness of 0.4 cm. Pelvic computed tomography confirmed a solid left ovarian tumor.

Hormone assays showed elevated serum LH (19.08 mIU/mL) and T (0.93ng/mL) levels (Table 1). The serum CA724 level was slightly abnormal at 15.86 IU/mL

(reference value: 0–6.00), but all other tumor marker levels were normal. The patient’s serum fasting blood glucose level was 8.84 mmol/L; and she was diagnosed with type 2 diabetes via oral glucose tolerance testing.

**Figure 4.**
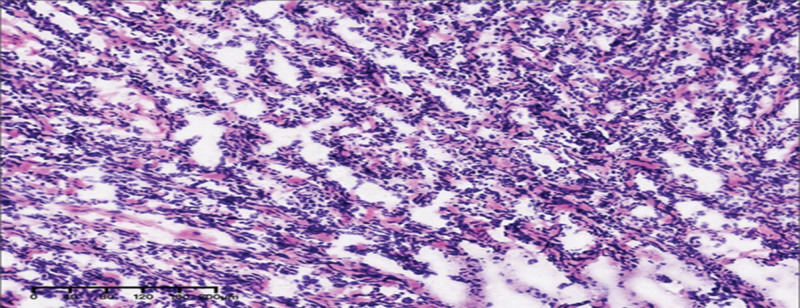
Pathological findings of the AGCT (mainly diffuse growth) in case B (25-year-old woman, left ovary). AGCT = adult-type granulosa cell tumor. (200×).

After obtaining the consent of the patient and family, we performed a laparoscopy. The left ovary had a solid mass 5 cm in diameter with an intact capsule. There was no evidence of extraovarian disease. A left ovarian cystectomy was performed. A rapid frozen pathology examination of the resected tumor indicated a GCT and the possibility of juvenile type. Fertility-sparing surgery with complete staging was performed with the consent of the patient’s family member. The final histopathological examination revealed an AGCT with diffuse growth (Fig. 4). Further staining revealed the presence of reticulated fibers (i.e., tumor cell nesting). The patient underwent 4 cycles of chemotherapy with paclitaxel and carboplatin as adjuvant treatments.

After treatment, the patient’s menstrual cycle returned to normal, and her serum LH and T levels returned to normal. There was no evidence of recurrence at the 11-month follow-up visit.

## 3. Discussion and literature review

Hyperandrogenemia can be attributed to the ovaries, adrenal glands, or the peripheral conversion of androgen precursors.^[5]^ The clinical manifestations of virilizing tumors are usually divided into defeminisation and virilization. Premenopausal women first experience oligomenorrhea or amenorrhea, followed by virilization.^[4]^ If hirsutism and virilization develop rapidly, especially with a high T level (usually >7 nmol/L) or pelvic mass, the possibility of an androgen-secreting tumor is high.^[6]^ In women with markedly elevated levels of T or its precursor androstenedione and normal levels of dehydroepiandrosterone, an androgen-secreting ovarian tumor is likely.^[4]^ However, androgen levels in some tumors may overlap with those observed in women with nonneoplastic hyperandrogenism. T levels in cases A and B were mildly elevated.

To the best of our knowledge, 5 reports have documented elevated LH levels in patients with adult-type ovarian GCTs (Table 2). As shown in Table 2, all previously reported patients were young (17–33 years of age) and had experienced secondary amenorrhea. The levels of FSH and E2 were either normal or low, whereas the levels of T were normal or high. The tumors were either cystic or solid. Our current cases involved women of childbearing age with the clinical symptoms of oligomenorrhea and secondary amenorrhea. Hormone assays revealed elevated LH and mildly elevated T levels, but normal FSH and E2 levels.

**Table 2:** Clinical characteristics and sex hormone level changes in patients with AGCTs with LH elevation based on a literature review.

Author	Year	Age at diagnosis	Tumor	LH (mIU/mL)	FSH (mIU/mL)	T (ng/mL)	E2 (pg/mL)
Nasu et al^[7]^	2007	28	Solid	High	Normal	Normal	Normal
Ran et al^[8]^	2017	33	Solid	High	Normal	Normal	Low
Gică et al^[9]^	2021	26	Cystic	High	Normal	Normal	Normal
Gu et al^[7]^	2022	31	Cystic	High	Normal	High	Normal
Kitajima et al^[10]^	2023	17	Cystic	High	Low	High	Normal

E2 = estradiol, FSH = follicle-stimulating hormone, LH = luteinizing hormone, T = testosterone

In the first case, an increase in serum LH level occurred earlier than that of T. One year after treatment for PCOS, A left ovarian tumor was found 1 year after treatment for PCOS. Oral contraceptives decreased the LH level to normal levels, which could confuse. This case suggests the importance of paying attention to increases in serum LH levels, which improves our understanding of early ovarian GCTs.

At present, the mechanism of LH elevation in AGCTs remains unclear. Immunohistochemistry has confirmed that the tumor cells are negative for LH.^[10,11]^ Some researchers have hypothesized that tumor cells produce factors that stimulate tumor is high.^[6]^ In women with markedly elevated levels of T or its precursor androstenedione and normal levels of dehydroepiandrosterone, an androgen-secreting ovarian tumor is likely.^[4]^ However, androgen levels in some tumors may overlap with those observed in women with nonneoplastic hyperandrogenism. T levels in cases A and B were mildly elevated.

To the best of our knowledge, 5 reports have documented elevated LH levels in patients with adult-type ovarian GCTs (Table 2). As shown in Table 2, all previously reported patients were young (17–33 years of age) and had experienced secondary amenorrhea. The levels of FSH and E2 were either normal or low, whereas the levels of T were normal or high. The tumors were either cystic or solid. Our current cases involved women of childbearing age with the clinical symptoms of oligomenorrhea and secondary amenorrhea. Hormone assays revealed elevated LH and mildly elevated T levels, but normal FSH and E2 levels.

In the first case, an increase in serum LH level occurred earlier than that of T. One year after treatment for PCOS, A left ovarian tumor was found 1 year after treatment for PCOS. Oral contraceptives decreased the LH level to normal levels, which could confuse. This case suggests the importance of paying attention to increases in serum LH levels, which improves our understanding of early ovarian GCTs.

At present, the mechanism of LH elevation in AGCTs remains unclear. Immunohistochemistry has confirmed that the tumor cells are negative for LH.^[10,11]^ Some researchers have hypothesized that tumor cells produce factors that stimulate the adenohypophysis to secrete a large amount of LH.^[11]^ LH secretion is regulated by both the hypothalamus and pituitary gland. The patient’s serum LH level decreased to normal after surgery but increased again upon recurrence,^[11]^ indicating that serum LH could serve as a tumor maker for AGCTs with LH elevation. This finding will be helpful in understanding the pathogenesis of androgen-secreting AGCTs in the ovary.

Normal ovarian granulosa cells secrete inhibin and AMH.^[3]^ Many studies have confirmed the utility of inhibin B and AMH as biochemical markers for the diagnosis, treatment effect evaluation, and monitoring of AGCTs recurrence.^[12–14]^ Increased serum AMH^[7,10]^ and inhibin B^[9]^ levels have been reported in AGCTs with elevated LH levels. The levels of AMH and inhibin B were both high in Case A and decreased to normal levels after surgery.

Surgery is the primary treatment for primary and recurrent AGCTs. If the disease is clinically confined to a single ovary in young women who wish to preserve their fertility, fertility-sparing surgery with complete staging is recommended. For all others, complete staging or cytoreductive surgery is recommended.^[15]^ For those with low-risk stage I disease, observation is preferred. However, for patients with intermediate- or high-risk stage I disease, observation or platinum-based chemotherapy is recommended. For patients in stages II to IV and/or those with disease recurrence, platinum-based chemotherapy should be administered.^[15]^

## 4. Conclusions

In cases of oligomenorrhea or secondary amenorrhea, an elevated serum LH level may be a sign of unknown tumors; thus, it is useful to evaluate the serum levels of inhibin B and AMH. Through early diagnosis and treatment, the prognosis is usually good and fertility can be preserved, especially in young patients with GCTs.

## Acknowledgments

We would like to express our gratitude to all those who helped us in the writing of this manuscript. We thank all peer reviewers for their opinions and suggestions.

## Author contributions

**Data curation:** Xiaodong Li, Yujing Wang.

**Formal analysis:** Xiaodong Li, Yujing Wang.

**Investigation:** Xiaodong Li, Yujing Wang.

**Methodology:** Xiaodong Li.

**Project administration:** Xiaodong Li.

**Resources:** Xiaodong Li, Chao Pang, Yuan Zhang.

**Visualization:** Xiaodong Li, Yujing Wang, Na Wang, Xuejiao Zhang, Zijie Fu.

**Writing—original draft:** Xiaodong Li, Yujing Wang, Na Wang, Xuejiao Zhang, Zijie Fu, Chao Pang, Yuan Zhang.

**Writing—review & editing:** Xiaodong Li, Yujing Wang.

**Conceptualization:** Yujing Wang.
